# Volumetric and textural analysis of PET/CT in patients with diffuse large B-cell lymphoma highlights the importance of novel MTVrate feature

**DOI:** 10.1097/MNM.0000000000001884

**Published:** 2024-08-02

**Authors:** Sándor Czibor, Zselyke Csatlós, Krisztián Fábián, Márton Piroska, Tamás Györke

**Affiliations:** a Department of Nuclear Medicine, Medical Imaging Centre, Semmelweis University,; b Faculty of Medicine, Semmelweis University and; c Mediso Medical Imaging Systems, Budapest, Hungary

**Keywords:** diffuse large B-cell lymphoma, machine learning, metabolic tumor volume, PET, texture analysis

## Abstract

**Objectives:**

To investigate the prognostic value of clinical, volumetric, and radiomics-based textural parameters in baseline [^18^F]FDG-PET/CT scans of diffuse large B-cell lymphoma (DLBCL) patients.

**Methods:**

We retrospectively investigated baseline PET/CT scans and collected clinical data of fifty DLBCL patients. PET images were segmented semiautomatically to determine metabolic tumor volume (MTV), then the largest segmented lymphoma volume of interest (VOI) was used to extract first-, second-, and high-order textural features. A novel value, MTVrate was introduced as the quotient of the largest lesion’s volume and total body MTV. Receiver operating characteristics (ROC) analyses were performed and 24-months progression-free survival (PFS) of low- and high-risk cohorts were compared by log-rank analyses. A machine learning algorithm was used to build a prognostic model from the available clinical, volumetric, and textural data based on logistic regression.

**Results:**

The area-under-the-curve (AUC) on ROC analysis was the highest of MTVrate at 0.74, followed by lactate-dehydrogenase, MTV, and skewness, with AUCs of 0.68, 0.63, and 0.55, respectively which parameters were also able to differentiate the PFS. A combined survival analysis including MTV and MTVrate identified a subgroup with particularly low PFS at 38%. In the machine learning-based model had an AUC of 0.83 and the highest relative importance was attributed to three textural features and both MTV and MTVrate as important predictors of PFS.

**Conclusion:**

Individual evaluation of different biomarkers yielded only limited prognostic data, whereas a machine learning-based combined analysis had higher effectivity. MTVrate had the highest prognostic ability on individual analysis and, combined with MTV, it identified a patient group with particularly poor prognosis.

## Introduction

Considered the most prevalent subtype of non-Hodgkin lymphomas, large B-cell lymphoma (DLBCL) is a clinically, pathologically, and molecularly heterogeneous haematological malignancy [1]. The usefulness of baseline 2-[^18^F]fluoro-2-deoxy-D-glucose (FDG) PET/computed tomography (PET/CT) scan in its initial clinical staging has accumulated substantial evidence and is included in current guidelines [2,3].

A number of FDG-PET/CT-based biomarkers have been recently identified that convey prognostic information beyond its inherent prognostic significance in defining the clinical stage of DLBCL. Among these metrics, metabolic tumor volume (MTV) and total lesion glycolysis (TLG) have demonstrated potential to contribute predictive value to established clinical scores, with the former being included in the novel international metabolic prognostic index (IMPI) [4–10]. When defining total body MTV, different segmentation algorithms can be used to identify lymphoma lesions as volumes of interest (VOIs) on PET series [11–16]. These VOIs can further be utilized for advanced texture analysis.

Texture analysis – or ‘radiomics’ – in medical imaging has a history of more than 50 years with Sutton and Hall being the pioneers in the field by investigating the possibility of automated classification of X-ray images in pulmonary diseases [17,18]. It is based on the evaluation of inter-voxel relationships and heterogeneity imperceivable to human eye and its utility has been widely researched in recent years both in CT and PET imaging [19–22].

Radiomics-based PET evaluations in the prognostic assessment of DLBCL have been scarce and heterogenous in approach, but showed promising results for further research [23–25].

We aimed to investigate the prognostic value of clinical, volumetric, and radiomics-based textural parameters individually and in a combined analysis with a machine learning algorithm.

## Methods

We retrospectively investigated baseline PET/CT scans of DLBCL patients performed at the Department of Nuclear Medicine of Semmelweis University’s Medical Imaging Centre. All patients received rituximab-based immunochemotherapy with a curative intent, mostly R-CHOP (rituximab combined with cyclophosphamide, doxorubicin, vincristine, and prednisolone). Only those patients were included who had at least 2 years of medical follow-up data available or showed documented disease progression within that time-frame.

At staging PET/CT, patients received 2.5–3.0 MBq/body weight kilogram FDG intravenously after a fasting period of more than 6 h. Sixty minutes after the injection of the radiopharmaceutical, a native CT scan and three-dimension PET-emission imaging were acquired with a hybrid PET/CT system (GE Discovery IQ5; GE Healthcare, Milwaukee, Wisconsin, USA). PET data was reconstructed using a novel Bayesian penalized likelihood-based algorithm with point-spread function (Q.Clear) and in some cases, with a conventional ordered subset expectation maximization (OSEM) algorithm (with six iterations and six subsets).

**Fig. 1:**
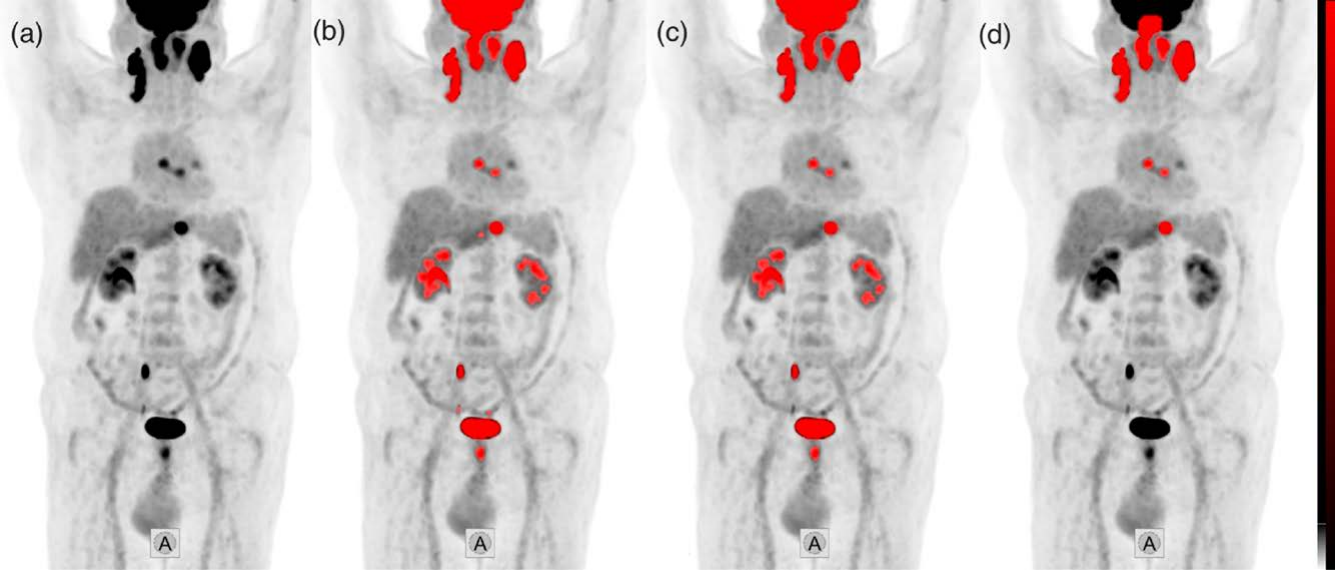
Segmentation algorithm of the lymphoma lesions on three-dimensional PET maximum intensity projection images; tumor delineations are indicated in red. (a) starting point; (b) automatic segmentation of voxels with SUV > 4.0; (c) elimination of volumes less than 1 ml; and (d) elimination of nonlymphomatous volumes.

Baseline PET images were evaluated by Mediso InterView Fusion software (Mediso Medical Imaging Systems, Budapest, Hungary) and lymphoma lesions were delineated using a fixed standardized uptake value (SUV)-based semiautomatic algorithm (SUV > 4.0 values) and were corrected manually when needed, to establish VOIs (Fig. 1). MTV was calculated as the sum of all lymphoma lesions’ volume on PET images, and TLG was determined as the sum of the product of each lesion’s metabolic volume and SUV_mean_.

MTVrate was introduced as the quotient of the largest lesion’s volume and total body MTV.

The VOI of the largest lymphoma lesion was used to extract first-, second-, and higher-order textural features, 44 in total. Patients with volume of the largest lymphoma lesion under 30 ml were excluded to avoid dependence on volume when calculating second-order entropy [26].

Receiver operating characteristics (ROC) analyses were performed to assess the prognostic performance of clinical and PET-based (volumetric and textural) values and to define optimal cutoff points. These cutoff points were used to determine low- and high-risk groups for each biomarker and the progression-free survival (PFS) of these groups was evaluated by log-rank analysis.

Where both OSEM and Q.Clear PET-reconstructions were available, lesion-segmentation was performed individually on both image sets and intraclass correlation coefficients (ICCs) were calculated.

Finally, a machine learning algorithm was used to build a prognostic model from the available clinical, volumetric, and textural data based on logistic regression. To avoid overfitting, we utilized elastic net regularization, the sum of L1 (Lasso) and L2 (Ridge) regularization functions. After preprocessing, repeated cross-validation was used to train the model in three cycles, splitting the patient population 70% : 30% into training and test sets (with reassigning after the first and second round). The model cleared the redundant parameters and gave the remaining ones relative importance. At the end, ROC analysis was performed with the model to determine its prognostic value.

Statistical analysis was performed using R (version 4.2.3) (R Foundation for Statistical Computing, Vienna, Austria) and SigmaPlot (version 11.0; Systat Software Inc., Chicago, Illinois, USA).

## Results

### Clinical and volumetric data

The final cohort encompassed 50 patients and Table 1 contains their demographic and clinical data.

**Table 1:** Patient characteristics and clinical data

Characteristic		*n* = 50 (100%)
Sex	Male	27 (54%)
	Female	23 (46%)
Age	Range	20–83
	Median	67
	>60 years	35 (70%)
Histopathologic status		
	GC	17 (34%)
	non-GC	33 (66%)
Stage		
	I	3 (6%)
	II	8 (16%)
	III	8 (16%)
	IV	31 (62%)
R-IPI		
	1	9 (18%)
	2	15 (30%)
	3	16 (32%)
	4	10 (20%)
LDH		
	Normal	17 (34%)
	Elevated	33 (66%)

GC, germinal center B-cell; LDH, lactate-dehydrogenase; R-IPI, Revised International Prognostic Index.

**Fig. 2:**
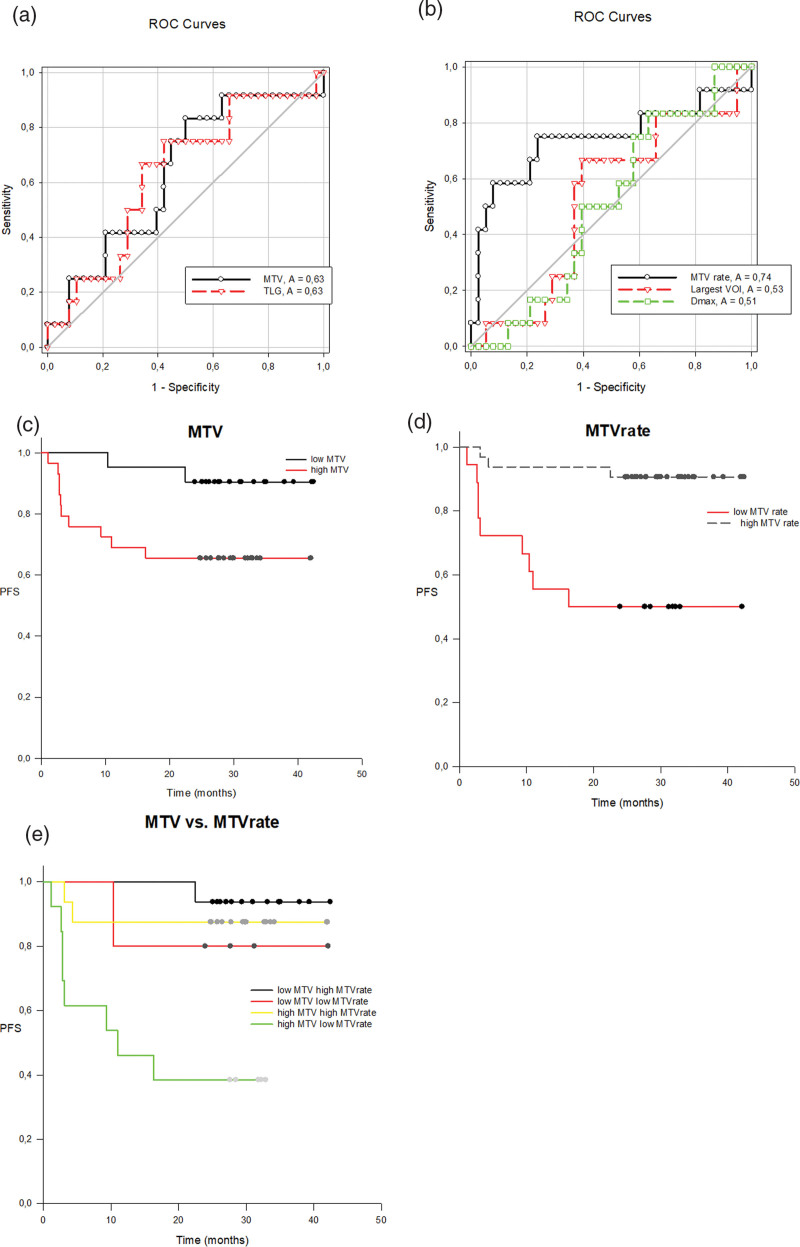
Receiver operating characteristic curves for 24-months PFS for (a) MTV and TLG; (b) MTVrate, maximum lesion volume (largest VOI), and Dmax. Kaplan–Meier curves of progression-free survival between low- and high-risk patient groups divided according to (c) MTV; (d) MTVrate; (e) MTV and MTVrate combined. Dmax, maximum lesion diameter; MTV, metabolic tumor volume; PFS, progression-free survival; TLG, total lesion glycolysis; VOI, volumes of interest.

Among clinical features, Ann-Arbor stage had an area-under-the-curve (AUC) of 0.60 and its dichotomic early/advanced classification did not show significant difference in PFS (*P* = 0.185); however, phenotypic status of the DLBCL had an impact on survival as patients with germinal center B-cell (GC) phenotype had a higher 24-months PFS than the non-GC group (94 vs. 67%; *P* = 0.038). Lactate-dehydrogenase (LDH) levels showed an AUC of 0.68, whereas MTV and TLG both had AUCs of 0.63 (Fig. 2). The ROC analysis-based optimal cutoff point for MTV was 378.5 ml and dividing patients into low- and high-risk groups along this value resulted in cohorts with significantly different 24-months PFS (94 vs. 68%; *P* = 0.036).

The highest AUC on ROC analysis of the clinical and volumetric parameters was given by the newly-introduced parameter of MTVrate, at the value of 0.74 and log-rank analysis supported its ability to provide patient groups with significantly different PFS (91 vs. 50%; *P* < 0.001) when dividing the whole population along the ROC-based optimum cutoff of 0.60 (Fig. 2).

Moreover, a combined survival analysis including MTV and MTVrate allowed to identify a subgroup with particularly low PFS at 38%, characterized by high MTV and low MTVrate (Fig. 2).

### Textural features

When investigating first-, second-, and higher-order textural features individually, the majority did not show significant prognostic value, with the only exception of the skewness parameter that had an AUC of 0.55 on ROC analysis and allowed to stratify two patient groups with significantly different PFS (68 vs. 94%; *P* = 0.046) on log-rank analysis.

Twenty-nine of the fifty included patients had two different PET-reconstructions (OSEM and Q.Clear) available. Among these parameters, first-order textural features all had an ICC over 0.9 while second- and higher-order textural features showed greater variance in ICC values, but were mainly above 0.8 as presented in Table 2.

**Table 2:** Intraclass correlation coefficients of second- and higher-order textural features between two different PET-reconstructions

ICC ≥ 0.9		
Textural feature	ICC	95% CI
Entropy	0.96	0.91–0.98
Homogeneity	0.96	0.93–0.98
Intensity variance	0.90	0.81–0.95
Skewness	0.91	0.82–0.96
Coarseness	0.92	0.83–0.96
Busyness	0.98	0.96–0.99
High gray-level zone emphasis	0.94	0.87–0.97
Short-zone high gray-level emphasis	0.93	0.85–0.96
Zone-length non-uniformity	0.90	0.81–0.95
Short-run emphasis	0.95	0.89–0.97
Long-run emphasis	0.94	0.88–0.97
Low gray-level run emphasis	0.92	0.83–0.96
High gray-level run emphasis	0.94	0.87–0.97
Short-run high gray-level emphasis	0.91	0.81–0.95
Long-run high gray-level emphasis	0.94	0.89–0.97
Run length non-uniformity	0.93	0.86–0.97

ICC < 0.9		
Textural feature	ICC	95% CI
Correlation	0.83	0.67–0.92
Contrast	0.87	0.73–0.93
Size variance	0.80	0.61–0.90
Kurtosis	0.74	0.51–0.87
Complexity	0.77	0.56–0.88
Short-zone emphasis	0.89	0.80–0.95
Long-zone emphasis	0.55	0.28–0.76
Low gray-level zone emphasis	0.81	0.63–0.91
Short-zone low gray-level emphasis	0.73	0.50–0.86
Long-zone low gray-level emphasis	0.73	0.50–0.86
Long-zone high gray-level emphasis	0.72	0.49–0.86
Gray-level non-uniformity	0.80	0.61–0.90
Zone percentage	0.81	0.64–0.91
Short-run low gray-level emphasis	0.66	0.39–0.82
Long-run low gray-level emphasis	0.88	0.77–0.94
Run percentage	0.74	0.53–0.87

CI, confidence interval; ICC, intraclass correlation coefficient.

### Combined analysis with machine learning

**Fig. 3:**
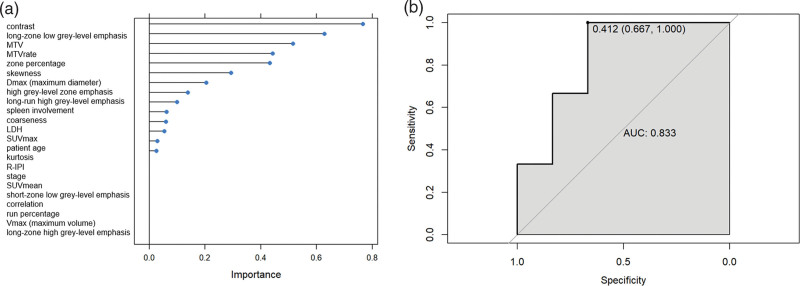
Results of the machine learning-based model for prognostic analysis: (a) importance of different parameters within the model; (b) receiver operating characteristic curve for 24-months progression-free survival.

In the machine learning-based model including clinical, volumetric, and textural values, the highest relative importance was attributed to the textural features contrast, long-zone low gray-level emphasis (LZLGE), zone percentage, and skewness while regarding volumetric features, both MTV and MTVrate remained in the model as important predictors of 24-months PFS. From clinical parameters, LDH levels, spleen involvement, and patient age had some lesser importance in the model. Implementing the model for prognosis evaluation the AUC on ROC analysis reached 0.83 (Fig. 3), and maximizing the sum of sensitivity and specificity on the ROC curve yielded a cutoff point with a sensitivity of 66.7%, and specificity of 100%

## Discussion

In this retrospective analysis of baseline PET data to assess prognosis in DLBCL patients, utilizing a machine learning-based combined analysis was highly efficacious, while individual assessment of various clinical, volumetric, and textural biomarkers produced limited prognostic data.

To our best knowledge, this is the first study to introduce MTVrate, the quotient of the volume of the greatest lesion and the total body MTV, and in our patient cohort, this value had the highest individual prognostic value among the investigated volumetric and clinical parameters (AUC = 0.74). Interestingly, the results indicated that worse prognosis correlated with lower MTVrates, pointing to that having more, relatively smaller, scattered lymphoma lesions was worse than a large lesion accompanied by few or no smaller ones. This value, as a ratio, does not take exact lymphoma lesion size in account; however, when combining MTVrate with total-body MTV, a patient group with a particularly poor 24-months PFS of 38% was identified.

In our patient cohort, MTV and TLG yielded only moderately promising prognostic performance and AUC on ROC analyses and even LDH level performed slightly better. Nevertheless, similar to several previous studies, ROC-optimum-based patient risk stratification of MTV resulted in groups with significantly different PFS [4–7].

Textural features are known to be influenced by several different technical factors, including the reconstruction algorithm [26,27]. In our subanalysis, first-order textural features all showed good correlation; however, we found that around half of the investigated second- and higher-order radiomic features had more different values between PET-reconstructions using either a more traditional OSEM or a relatively novel, Bayesian penalized likelihood-based algorithm with point-spread function (Q.Clear).

When analysing textural features individually, only skewness had a significant differentiating power in prognostic evaluation. The combined, machine learning-based model encompassed mainly second- and higher-order textural features as important prognostic biomarkers, as well as both MTV and MTVrate with some clinical parameters also carrying some lesser relevance. This model had a great prognostic ability with an AUC of 0.83 on ROC analysis.

In 2020, Aide *et al.* conducted a study on a sample of 132 patients, examining the individual prognostic power of conventional and textural parameters. Patients were divided into teaching and validation groups in a ratio of 105 : 27 [23]. In ROC analysis, among the higher-order variables long-zone high gray-level emphasis (LZHGE) yielded the highest AUC at 0.69, it demonstrated significant differentiation ability at log-rank analysis and was the only independent predictor of for 24-months PFS in Cox survival analysis. Furthermore, in log-rank analysis, MTV, four second-order, and five higher-order parameters (including LZHGE) showed differentiating power for 24-months PFS.

In 2021, Coskun *et al.* used similar methods to train a prediction model to estimate patient outcomes on a sample size similar to our own [24]. They studied a sample of 45 patients and used elastic net regularization logistic regression for model building, trained with three-fold cross-validation. The essential difference of the study from ours was the preparation of the study sample. VOIs were determined according to a SUV_max_ > 40% criterion and the parameters to be tested were extracted from the largest six (or as many as the patient had if less) lesions of each patient, going by texture and SUV-based indicators. As a result of their research, they found that SUV_max_ and gray-level co-occurance matrix (GLCM) dissimilarity were independent predictors of 24-months PFS, and their model showed an AUC of 0.81 in ROC analysis. It is interesting to note that in a similar sample size, with similar model design, despite the different data collection procedure, our prediction accuracy was found to be similar.

Eertink *et al.* recently analyzed baseline PET scans of 317 newly diagnosed DLBCL patients and found that a model encompassing tumor- [MTV, SUV_peak_, and Dmax_bulk_ (distance between the two lesions that were furthest apart)] and patient-related parameters (WHO performance status and age >60 years) had the best prognostic ability with an AUC of 0.79 [25]. These results were validated on a larger, pooled cohort [28].

The present study has some limitations. Firstly, our analysis was retrospective in nature, however, with the inclusion of consecutive patients undergoing baseline PET/CT at our department, the data could resemble real-life practice. Secondly, the relatively low patient number and the abundance of different investigated clinical, volumetric, and textural biomarkers posed the danger of the overfitting of our machine learning-based model, for which we tried to compensate with the implementation of elastic net regularization. Thirdly, as more evidence is gathered on the importance of the molecular heterogeneity of DLBCL, our lack of molecular genetic data lessened the strength of our combined model [29–31].

### Conclusion

Our retrospective investigation of baseline PET data to evaluate prognosis of patients with DLBCL highlighted that individual evaluation of different clinical, volumetric, and textural biomarkers yielded only limited prognostic data, whereas a machine learning-based combined analysis had high effectivity. Furthermore, the newly defined MTVrate (as the quotient of the largest lesion’s volume and total body MTV) had the highest prognostic ability on individual analysis and, combined with MTV, it allowed to identify a patient group with particularly poor prognosis.

## Acknowledgements

K.F. is an employee of Mediso Medical Imaging Systems Ltd.

### Conflicts of interest

There are no conflicts of interest.
